# Incidence Rates of Adverse Events (CTCAE Severity ≥3) During HER2-Directed Breast Cancer Treatment Assessing the Impact of App-Based Electronic Patient-Reported Outcomes in the Randomized Controlled PRO2 Trial

**DOI:** 10.3390/curroncol33100618

**Published:** 2026-10-14

**Authors:** Andreas Trojan, Marion van Mackelenbergh, Cosima Brucker, Helmut Forstbauer, Markus Thiel, Hans-Christian Kolberg, Manfred Welslau, Janine Kreiss-Sender, Stefan Dröse, Sven Roth, Philip Raeth, Marcus Vetter, David Blum, Peter A. Fasching

**Affiliations:** 1 Brustzentrum Zürichsee, Seestrase 80, 8810 Horgen, Switzerland; 2 Department of Clinical Pharmacology and Toxicology, University Hospital Zurich, 8008 Zurich, Switzerland; 3 Department of Gynecology and Obstetrics, Schleswig-Holstein University Hospital, Kiel Campus, 24105 Kiel, Germany; mariontina.vanmackelenbergh@uksh.de; 4 Department of Gynecology and Obstetrics, Klinikum Nürnberg, University Hospital of Paracelsus Medical University, 90419 Nuremberg, Germany; cosima.brucker@klinikum-nuernberg.de; 5 Practice Network Troisdorf, 53840 Troisdorf, Germany; forstbauer@onkologie-rheinsieg.de; 6 Practice Network for Hematology and Oncology, 66822 Lebach, Germany; markusthiel@onkologie-lebach.de; 7 Knappschaft Kliniken Marienhospital Bottrop, 46236 Bottrop, Germany; hans-christian.kolberg@mhb-bottrop.de; 8 Oncology Aschaffenburg, 63739 Aschaffenburg, Germany; manfred.welslau@klinikum-ab-alz.de; 9 Gynecological Oncology Practice, 38100 Braunschweig, Germany; kreiss-sender@frauenaerzte-casparistr.de; 10 Palleos Healthcare GmbH, 65189 Wiesbaden, Germany; stefan.droese@palleos.com (S.D.); philip.raeth@palleos.com (P.R.); 11 Spital Zollikerberg, 8125 Zollikerberg, Switzerland; sven.roth@spitalzollikerberg.ch; 12 Center of Oncology & Hematology, Cancer Center Baselland, Kantonsspital Baselland, 4410 Liestal, Switzerland; marcus.vetter@ksbl.ch; 13 Department of Radiation Oncology, University Hospital Zurich, 8008 Zurich, Switzerland; david.blum@usz.ch; 14 Department of Gynecology and Obstetrics, University Hospital Erlangen, Comprehensive Cancer Center Erlangen-EMN (CCC ER-EMN), 91054 Erlangen, Germany; peter.fasching.studien@uk-erlangen.de

**Keywords:** electronic patient-reported outcomes (ePRO), HER-directed breast cancer therapy, digital health app, adverse events (AEs), emergency consultation, hospitalization

## Abstract

(1) Background: the evaluation of app-based ePROs is increasingly being used in clinical trials with cancer patients while a medical effect for patients is a matter of controversy; (2) Methods: in the randomized (1:1) controlled PRO2 trial the effect of the medidux^TM^ app on the rate of “high-grade AEs” (HAEs, CTCAE severity ≥3) within patients receiving HER2-directed breast cancer treatment (arm A) was compared to a control-group that did not use the app (arm B); (3) Results: Among patients with observed (non-imputed) HAE data (*n* = 180 of *N* = 189 in the ITT population), the rates of documented HAEs were 21.51% in arm B and 17.24% in arm A. Logistic regression of HAE incidence in the full ITT population (*N* = 189), using reference-based multiple imputation for missing values, showed a positive tendency towards app use, but no significant difference (odds ratio arm (A/B): 0.797, 95% CI: 0.363, 1.752; *p*-value 0.569). Also, AE-related secondary endpoints “emergency consultations” (risk ratio arm (A/B): 0.842, 95% CI: 0.464, 1.522; *p*-value 0.570) and “hospitalizations” (risk ratio arm (A/B): 0.575, 95% CI: 0.237, 1.329; *p*-value 0.201) had no significant differences in negative binomial regression. (4) Conclusions: Although the effect on HAE rates was not statistically significant, intensive app use was observed and adverse events recorded by participants corresponded well with the known drug-side-effect-profiles. These exploratory findings suggest a potential supporting role of the app during out-patient cancer treatment, which needs confirmation in adequately powered studies.

## Simple Summary

The use of a digital health application (app) by cancer patients enables the daily, structured, and standardized recording of symptoms and side effects of cancer medication. This not only facilitates communication between the patient and their treating physician but could also lead to the early detection of adverse side effects and thus may have a clinical impact. To investigate this, a group of HER2-positive breast cancer patients being treated with HER2-targeted medication used the medidux™ app for 12 weeks, and all adverse effects with a defined severity level, as well as hospitalizations and unplanned emergency visits, were recorded. A comparison with a control group of equal size that did not use the app showed a tendency toward a reduction in these recorded parameters in the app-using group; however, the results were not statistically significant.

## 1. Introduction

Electronic patient-reported outcomes (ePROs) are being applied with growing frequency in oncology clinical research [1], allowing symptom and outcome data to be captured in a structured, standardized manner as part of patients’ daily lives. Aggregating and analyzing anonymized information across large numbers of patients, together with collaboration among (research) centers, can generate a foundation for improving both the quality and efficiency of care. Beyond facilitating communication, however, app-based ePRO tools may offer patients additional benefits. Cancer patients receiving systemic therapy frequently experience treatment-related side effects, most commonly fatigue (80%), pain (48%), and nausea/vomiting (48%) [2]. Both the type of therapy administered and the patient’s performance status have been linked to the likelihood of hospitalization [3]. An estimated 35% of patients newly diagnosed with cancer experience an unplanned hospitalization, with 67% of those hospitalized having already presented to the emergency department beforehand [4]. Among 154 patients with colorectal cancer, 28% of hospital admissions were attributable to complications of cancer treatment, and 19% of these were judged potentially avoidable [5]. In a further colorectal cancer cohort, the majority of unplanned consultations (72%) took place within 30 days after the most recent chemotherapy session, and 10% of these visits led to hospitalization [6]. In a retrospective analysis of unplanned hospital admissions among outpatients, 74% had received chemotherapy within the preceding 6 months, and 69.7% of these admissions occurred within 4 weeks of chemotherapy administration. Recent studies support the notion that structured, ongoing recording of patient-reported symptoms enables earlier detection of adverse events related to cancer therapy, and that timely action in response can help prevent progression to more severe grades. Compared with control groups, digital app- or web-based systems for symptom monitoring have been shown to lower rates of emergency admissions and hospitalizations, with some studies additionally reporting extended (progression-free) survival [7,8,9,10]. In two of these studies, use of the digital application was further associated with a reduction in SAEs relative to controls [8,11]. In the PRO study, patients with cancer undergoing systemic therapy in a (neo-)adjuvant or non-curative setting self-assessed their side effects using the consilium care™ app over a 90-day observation period [12]. Unplanned (emergency) consultations and hospitalizations occurring during this period were recorded by the treating physicians, and their incidence was benchmarked against retrospective data from a matched patient cohort at two Swiss tumor centers. A multivariate regression model showed a significant interaction (*p* = 0.048) between the “consilium group” and the chemotherapy factor [12], which prompted a subgroup analysis pointing to a meaningful risk reduction for the intervention group among chemotherapy patients—reflected in an odds ratio of 0.53, equivalent to roughly half the risk for patients in the “consilium group”. However, the analysis was limited by the relatively heterogeneous patient population and the comparison with retrospective data. In the subsequent follow-up controlled randomized study PRO2 described here, data was collected in a clearly defined patient group, HER2-positive breast cancer patients whose HER2-targeted therapy included a chemotherapy component. The intervention group received the improved app successor version medidux™, while the control group did not use an app. “High-grade adverse events” (HAEs, defined as AEs with a CTCAE grade >2), which incidences were used to assess a possible medical benefit of the app, were evaluated by a blinded adjudicator in order to exclude bias as far as possible. Supportive secondary analyses recorded unplanned emergency consultations and hospitalizations associated a priori with AEs, the influence of the app on therapy adherence, adherence to app use, and AEs self-reported by patients in the intervention group.

## 2. Materials and Methods

### 2.1. Study Population

This study was conducted at 23 gynecological and oncological clinics and practices in Germany and 5 in Switzerland. Patients eligible for an approved HER2-targeted therapy were enrolled, comprising both HER2-positive breast cancer (IHC 3+, or IHC 2+ with a positive in situ hybridization result [ISH+]) and HER2-low breast cancer according to the current HER2-low definition (IHC 1+, or IHC 2+ with a negative ISH result [ISH-]), irrespective of hormone receptor status. Although patients with HER2-low breast cancer were eligible per protocol, none were ultimately enrolled; all 189 patients included in the ITT population were HER2-positive by FISH (Table 1). Patients started neoadjuvant, adjuvant, or palliative chemotherapy in combination with HER2-targeted therapy (including tyrosine kinase inhibitors [TKIs]) or an antibody-drug conjugate (ADC) between August 2022 and June 2025. The inclusion criteria included (1) adult patients aged >18 years with a signed declaration of consent, (2) ECOG performance status ≤ 1, (3) Sufficient command of the German language as assessed by the investigator and (4) Presence of a personal smartphone with iOS or Android system. Patients who had already used the medidux™ (version 3.2; Zurich, Switzerland) app or its predecessor consilium care™ before enrolling in the study were excluded. The study had to be terminated prematurely. Recruitment was stopped early in February 2025 after 194 patients had been enrolled. The study is registered on ClinicalTrials.gov (NCT05425550).

### 2.2. Study Design

This was a multicenter, prospective, randomized and controlled study to evaluate the medidux™ app during an observation period of 12 weeks (max. 16 weeks in case of postponements in the initially planned therapy). Breast cancer patients receiving a HER2-targeted therapy were randomized 1:1, electronically via an eCRF system administered by a contract research organization (CRO), with blinded allocation communicated to the treating physician, into an intervention group (arm A) that was supplied with the medidux™ app on their personal smart phone and a control group (arm B) that did not use the app. Treatment intention (curative vs. palliative) was not used as a stratification factor; it was included as a baseline covariate in the adjusted analysis of the primary endpoint. For the analysis of the primary study endpoint, investigators documented all AEs, either SAE type or AE type with CTCAE severity ≥3, for each patient, with start date within the observation period. In the following, these events are referenced as high-grade AEs (HAE). Events classified as “hair loss” (alopecia) were excluded from the analysis. Adverse events were mapped to the hierarchy and thesaurus terms of the Medical Dictionary for Regulatory Activities (MedDRA, latest version). Severity was determined according to NCI CTCAE v5.0. In addition to the initial assessment by the investigators and a medical monitor, the HAEs were assessed by a medical expert (adjudicator) who was blinded to treatment allocation, and a classification was made independently of the previous assessment, based on the events and symptoms documented by the investigator. For the primary endpoint, HAEs were defined and confirmed based on documentation by the investigator during scheduled study visits, not directly on symptoms self-reported by patients via the app. In cases where the blinded adjudicator’s classification differed from the initial assessment by the investigator and medical monitor, the adjudicator’s independent classification was considered decisive and used for the primary endpoint analysis. Secondary endpoints included data on unscheduled emergency visits, hospitalizations, dose reductions in chemotherapy or antibody-drug conjugates, and for patients who used the app (arm A): number and severity of self-reported ePRO symptoms and app usage (adherence).

### 2.3. Data Collection

Clinical patient data including AE and SAE recording and baseline characteristics were documented in an eCRF and confirmed by the participating investigators. Toxicity assessments were performed at protocol-defined visits, occurring at intervals of 3 weeks, according to an identical schedule in both study arms. The medidux™ smartphone app was used for the collection of toxicity data by patients in the intervention group. medidux™ is a CE-certified software application that enables the monitoring of well-being and symptoms of patients undergoing cancer treatment. medidux™ creates symptom progression charts based on patient input and notifies the patient to contact the treatment team if symptoms are outside the acceptable range as defined by the Common Terminology Criteria for Adverse Events (CTCAE) standards. In the app, patients selected a symptom from a predefined catalogue and rated its severity on a 0–10 visual analogue scale, supported by symptom-specific descriptions of each severity level; ratings were mapped to severity grades in fixed two-point bands (2.1–4.0: grade 1; 4.1–6.0: grade 2; 6.1–8.0: grade 3; 8.1–10.0: grade 4), and ratings above grade 2 prompted the app to advise the patient to contact the treatment team. This patient-facing instrument has not undergone formal validation; self-reported symptom data were used descriptively and did not contribute to the primary endpoint, which was graded by the investigators according to CTCAE v5.0. Patients were advised via the app to contact their care team on their own initiative following an alert; the care team was not automatically notified. medidux™ improves communication between the patient and the physician through the structured and standardized recording of Patient Reported Outcomes (PROs). For more information see [13].

### 2.4. Statistical Analysis

The analysis of the primary endpoint was performed on the intent-to-treat (ITT) population of all randomized patients using a logistic regression model with correction for relevant baseline covariates. The *p*-value of a 2-sided likelihood ratio test for significance of the treatment group (5% significance level) and the corresponding odds ratio with 95% confidence interval (profile likelihood) are reported. In addition, the incidence rates in the two treatment groups are reported with 95% confidence interval (Clopper-Pearson). Missing values were treated in the primary analysis using (reference-based) multiple imputation.

Further descriptive statistics are reported for incidence proportions according to the MedDRA-coded “Preferred Terms”, sorted by system-organ class, stratified by treatment group, to allow a qualitative consideration of differences between the arms with regard to individual AEs.

During the data review meeting, protocol deviations (PDs) were identified relating to inclusion/exclusion criteria with regard to timing of medical therapy start date, randomization date, and start of app intervention. This led to the exclusion of 4 participants in arm A and 1 in arm B from the ITT-population, and additionally 8 participants in arm A and 5 in arm B from the per-protocol (PP) population.

## 3. Results

### 3.1. Participant Disposition

**Figure 1:**
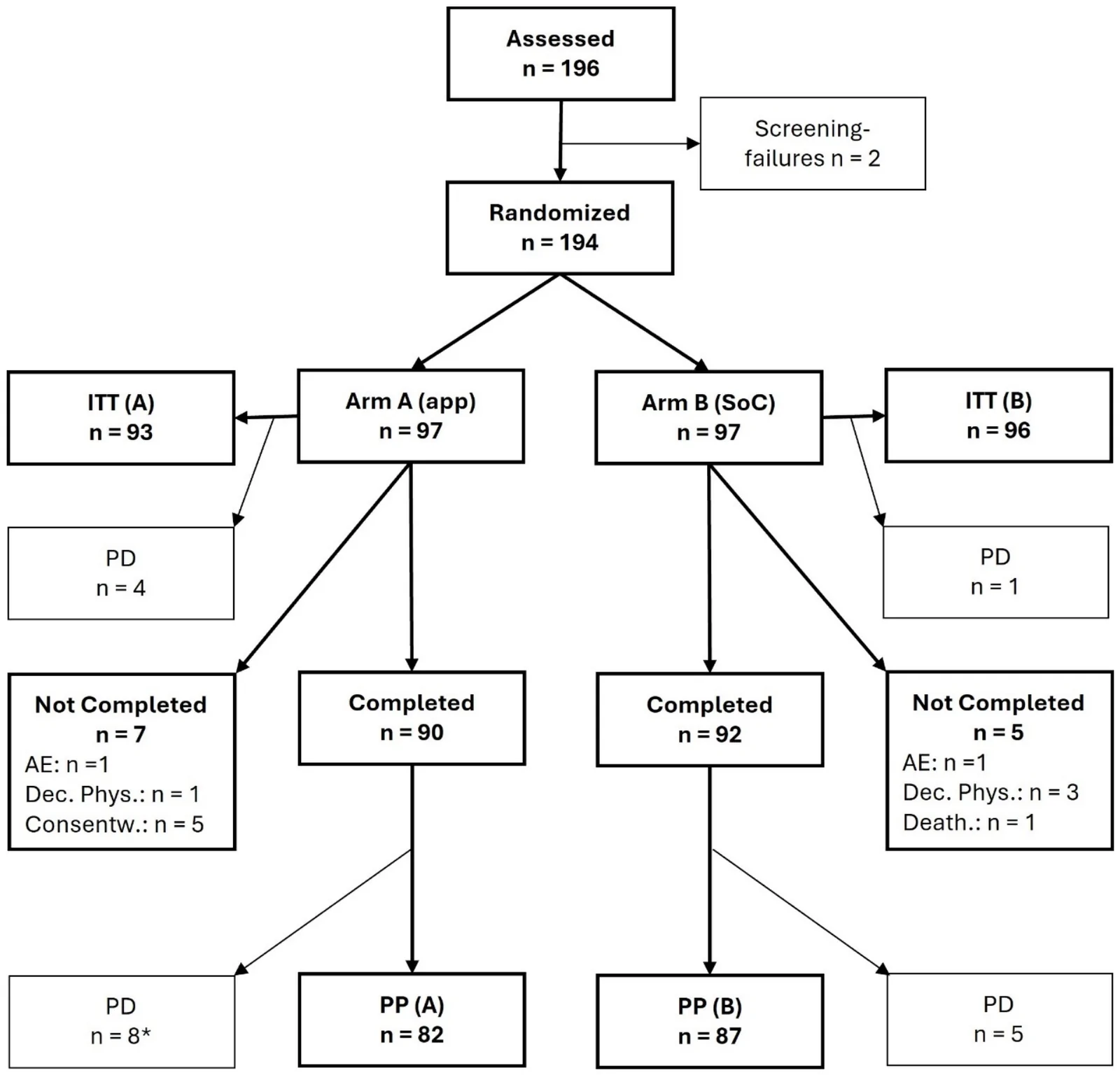
Consort diagram. Patients randomized into arm A received the medidux™-app, Patients in arm B constituted the control group, who did not receive the app. ITT: Intention-to-treat; SoC: Standard of Care; PD: Protocol Deviation; AE: Adverse Event; Dec. Phys.: Decision of Physician; Consentw.: Consent withdrawn; PP: Per Protocol. * There were actually 9 PDs that would have led to the exclusion of a patient from the PP population, but 1 patient was already excluded, because she did not complete the 12-week treatment.

Due to the premature termination of the study because of slow recruitment, only 194 participants (97 in the intervention arm A and 97 in the control arm B) could be included in the study. Of these, 7 participants in the intervention arm A and 5 participants in the control arm B discontinued the study due to various reasons as outlined in the Consort diagram (Figure 1).

### 3.2. Baseline Characteristics

All included participants were female with a mean age at inclusion of 52.4 years. Sociodemographic characteristics at baseline are presented in Table 1. Both groups were very similar with regard to the parameters of age, HER2 status, progesterone receptor status, distribution of tumor sizes, metastatic spread, breast cancer status, therapy intent and therapy type. With respect to treatment type, 87 patients (93.55%) in arm A and 91 (94.79%) in arm B received chemotherapy plus HER2-targeted therapy, while 6 patients (6.45%) and 5 patients (5.21%), respectively, received an antibody-drug conjugate (Table 1). There were smaller differences between the two groups with regard to the proportion of estrogen receptor status and the distribution of nodal statuses. Also, the observation time of the participants was comparable in both arms (Table S1) and corresponded on average to the 12 weeks specified by the protocol. As many as 93.7% of all participants completed all 5 visits specified in the protocol.

**Table 1:** Baseline characteristics ITT population.

Parameter	Level	Statistic	Arm A	Arm B	Total
Age	(all)	N (missing)	93 (0)	96 (0)	189 (0)
		Mean	52.45	52.32	52.39
		Std	10.725	11.805	11.257
		Min	26	26	26
		Q1	45	45	45
		Median	53	52	53
		Q3	60	62	60
		Max	80	84	84
		IQR	15	17	15
Sex	(all)	N (missing)	93 (0)	96 (0)	189 (0)
	F	N (%)	93 (100%)	96 (100%)	189 (100%)
HER2 Status (FISH) *	(all)	N (missing)	93 (0)	96 (0)	189 (0)
	NEGATIVE	N (%)	0 (0%)	0 (0%)	0 (0%)
	POSITIVE	N (%)	93 (100%)	96 (100%)	189 (100%)
Estrogen Receptor Status	(all)	N (missing)	93 (0)	96 (0)	189 (0)
	NEGATIVE	N (%)	35 (37.63%)	44 (45.83%)	79 (41.8%)
	POSITIVE	N (%)	58 (62.37%)	52 (54.17%)	110 (58.2%)
Progesterone Receptor Status	(all)	N (missing)	93 (0)	96 (0)	189 (0)
	NEGATIVE	N (%)	49 (52.69%)	55 (57.29%)	104 (55.03%)
	POSITIVE	N (%)	44 (47.31%)	41 (42.71%)	85 (44.97%)
Tumor Size (c or p) **	(all)	N (missing)	93 (0)	96 (0)	189 (0)
	NOT DONE	N (%)	1 (1.08%)	2 (2.08%)	3 (1.59%)
	T0	N (%)	1 (1.08%)	0 (0%)	1 (0.53%)
	T1	N (%)	6 (6.45%)	6 (6.25%)	12 (6.35%)
	T1A	N (%)	1 (1.08%)	3 (3.12%)	4 (2.12%)
	T1B	N (%)	7 (7.53%)	8 (8.33%)	15 (7.94%)
	T1C	N (%)	18 (19.35%)	21 (21.88%)	39 (20.63%)
	T2	N (%)	51 (54.84%)	50 (52.08%)	101 (53.44%)
	T3	N (%)	5 (5.38%)	3 (3.12%)	8 (4.23%)
	T4	N (%)	2 (2.15%)	2 (2.08%)	4 (2.12%)
	T4B	N (%)	0 (0%)	1 (1.04%)	1 (0.53%)
	T4D	N (%)	1 (1.08%)	0 (0%)	1 (0.53%)
Nodal Status (c or p) **	(all)	N (missing)	93 (0)	96 (0)	189 (0)
	N0	N (%)	49 (52.69%)	51 (53.12%)	100 (52.91%)
	N1	N (%)	33 (35.48%)	21 (21.88%)	54 (28.57%)
	N1A	N (%)	3 (3.23%)	4 (4.17%)	7 (3.7%)
	N1MI	N (%)	0 (0%)	1 (1.04%)	1 (0.53%)
	N2	N (%)	2 (2.15%)	6 (6.25%)	8 (4.23%)
	N2A	N (%)	0 (0%)	0 (0%)	0 (0%)
	N3	N (%)	1 (1.08%)	3 (3.12%)	4 (2.12%)
	NOT DONE	N (%)	2 (2.15%)	3 (3.12%)	5 (2.65%)
	NX	N (%)	3 (3.23%)	7 (7.29%)	10 (5.29%)
Metastatic Spread	(all)	N (missing)	93 (0)	96 (0)	189 (0)
	M0	N (%)	75 (80.65%)	74 (77.08%)	149 (78.84%)
	M1	N (%)	12 (12.9%)	15 (15.62%)	27 (14.29%)
	MX	N (%)	5 (5.38%)	7 (7.29%)	12 (6.35%)
	NOT DONE	N (%)	1 (1.08%)	0 (0%)	1 (0.53%)
Breast Cancer Status	(all)	N (missing)	93 (0)	96 (0)	189 (0)
	EARLY BREAST CANCER	N (%)	73 (78.49%)	76 (79.17%)	149 (78.84%)
	LOCALLY ADVANCED BREAST CANCER	N (%)	8 (8.6%)	5 (5.21%)	13 (6.88%)
	METASTATIC BREAST CANCER	N (%)	12 (12.9%)	15 (15.62%)	27 (14.29%)
Therapy Intent	(all)	N (missing)	93 (0)	96 (0)	189 (0)
	CURATIVE	N (%)	82 (88.17%)	84 (87.5%)	166 (87.83%)
	PALLIATIVE	N (%)	11 (11.83%)	12 (12.5%)	23 (12.17%)
Therapy Type	(all)	N (missing)	93 (0)	96 (0)	189 (0)
	CHEMOTHERAPY + HER2-TARGETED THERAPY (INCLUDING TKI)	N (%)	87 (93.55%)	91 (94.79%)	178 (94.18%)
	THERAPY WITH AN ANTIBODY-DRUG CONJUGATE	N (%)	6 (6.45%)	5 (5.21%)	11 (5.82%)

* Fluorescence in situ hybridization. ** Clinical staging or pathological staging.

### 3.3. Incidence of HAEs (CTCAE Severity ≥3)

The HAE incidence proportion in the ITT population (no imputation applied) were comparable in both study arms, with a slightly higher incidence in arm B than in arm A (Table 2).

The summary statistics for the incidence proportions of the preferred terms coded by MedDRA, sorted by system organ class and stratified by treatment group, also showed no major differences between the treatment groups (Table S2). In a logistic regression model that corrected for the important baseline covariates age, treatment intention (curative vs. palliative) and therapy type (CTX with targeted HER2 antibody vs. antibody-drug conjugate) and using reference-based multiple imputation of missing values, no significant differences were observed between the intervention arm A and the control arm B (Table 3). Only age showed a significant effect on the occurrence of HAEs, with a slight increase with increasing age. The corresponding odds ratio (A/B) for the proportion of HAEs results in a slightly reduced risk for arm A (0.797). The measured treatment effect was a 3.9 percentage-point absolute reduction in HAE incidence, corresponding to a relative reduction of 18.3%, with an adjusted OR of 0.797. However, the analysis showed a wide 95% CI and a non-significant *p*-value.

A sensitivity analysis of the primary endpoint with the PP population (Table S3) yielded a very similar result to the analysis with the ITT population (Table S3). The odds ratio of 0.866 calculated using logistic regression was also not significant (*p* = 0.724). When considering only SAE, a higher incidence is seen in the control arm (B) (Table S4). However, logistic regression does not show a significant odds ratio (Table S5).

### 3.4. Cumulative Incidence of the Occurrence of the First HAE and App Effect on Treatment Adherence

**Figure 2:**
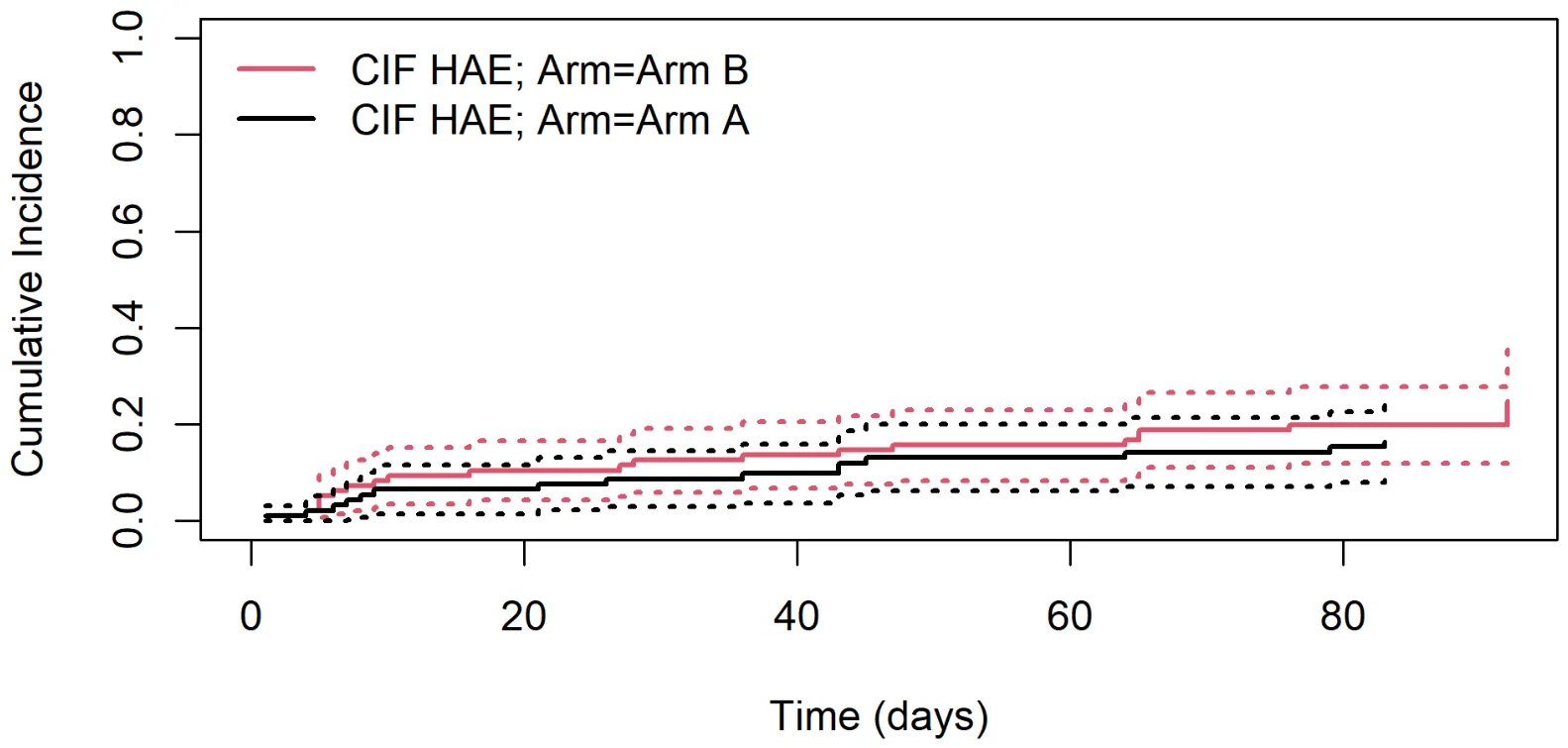
Cumulative incidence function (CIF) estimate (Aalen-Johansen) for time to first HAE. Analysis was performed with the ITT population. The dashed lines indicate 95% CIs.

The analysis of the cumulative incidence of the occurrence of the first HAE shows an almost identical course for intervention arm A and control arm B (Figure 2). It is interesting to note that in both arms almost half of the reported first HAEs occurred within the first 21 days after the start of the therapy.

When evaluating the data, it was important to determine whether using the app had an impact on therapy adherence or led to a greater reduction in medication dosage. The PP population was chosen for analysis since patients with early study discontinuation could have unobserved changes to therapy post-discontinuation and patients with no app adherence cannot have an app-induced dose reduction. Analysis focused on the observable 12-week intervention period. The use of the medidux™ app did not lead to a reduction in chemotherapy or antibody-drug-conjugate therapy adherence (Figure S1). The incidence of CTX dose reductions in the PP population was slightly higher in arm A (Table S6). In the logistic regression analysis, the covariate “Treatment Intention” was removed as no patients in palliative setting had dose reductions. Anyway, the determined odds ratio (A/B) was not significant (Table S7).

### 3.5. Incidence Rates of Emergency Consultations and Hospitalizations

As supportive analyses, the incidence rates of emergency consultation and hospitalization of the ITT-population were determined. The incidence of emergency consultations was similar in both arms (Table 4), the risk ratio (arm A/B) estimated by negative binominal regression was not significant (Table S8). Participants of arm A showed a slightly lower risk for hospitalizations (Table 4), but the negative binominal regression was also not significant (Table S9).

### 3.6. ePRO Symptoms Profile

**Figure 3:**
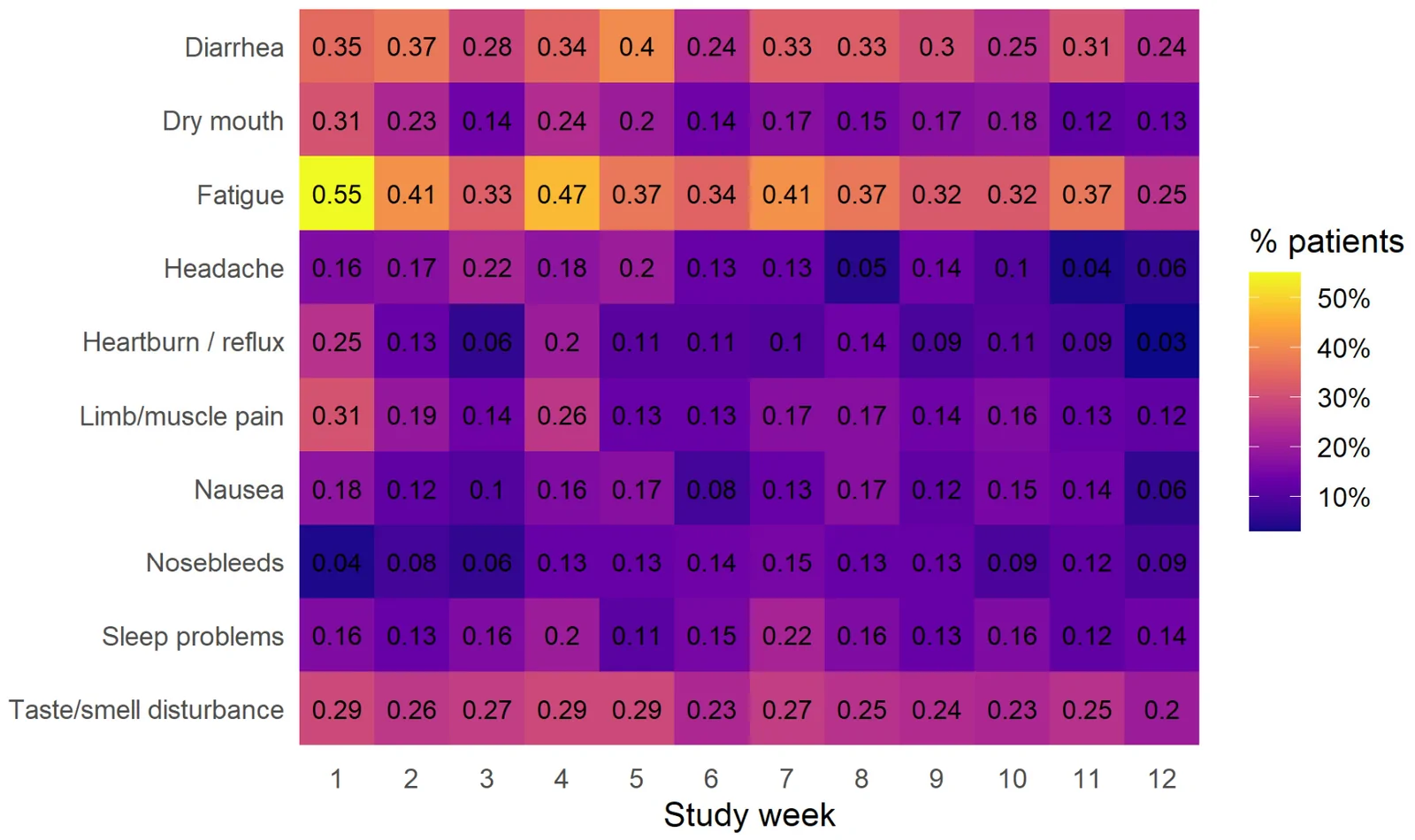
Weekly ePRO-CTCAE Incidence (top 10 Symptoms, full ITT Population Arm A).

Symptoms documented by participants in arm A in the medidux™ app with an incidence of over 50% included fatigue (79.6%), diarrhea (72.0%), limb/muscle pain (57.0%) and taste/odor disturbances (55.9%). An evaluation of the 10 most common side effects documented by the participants shows that these generally decrease over the course of the 12-week observation period (Figure 3).

### 3.7. App Adherence

**Figure 4:**
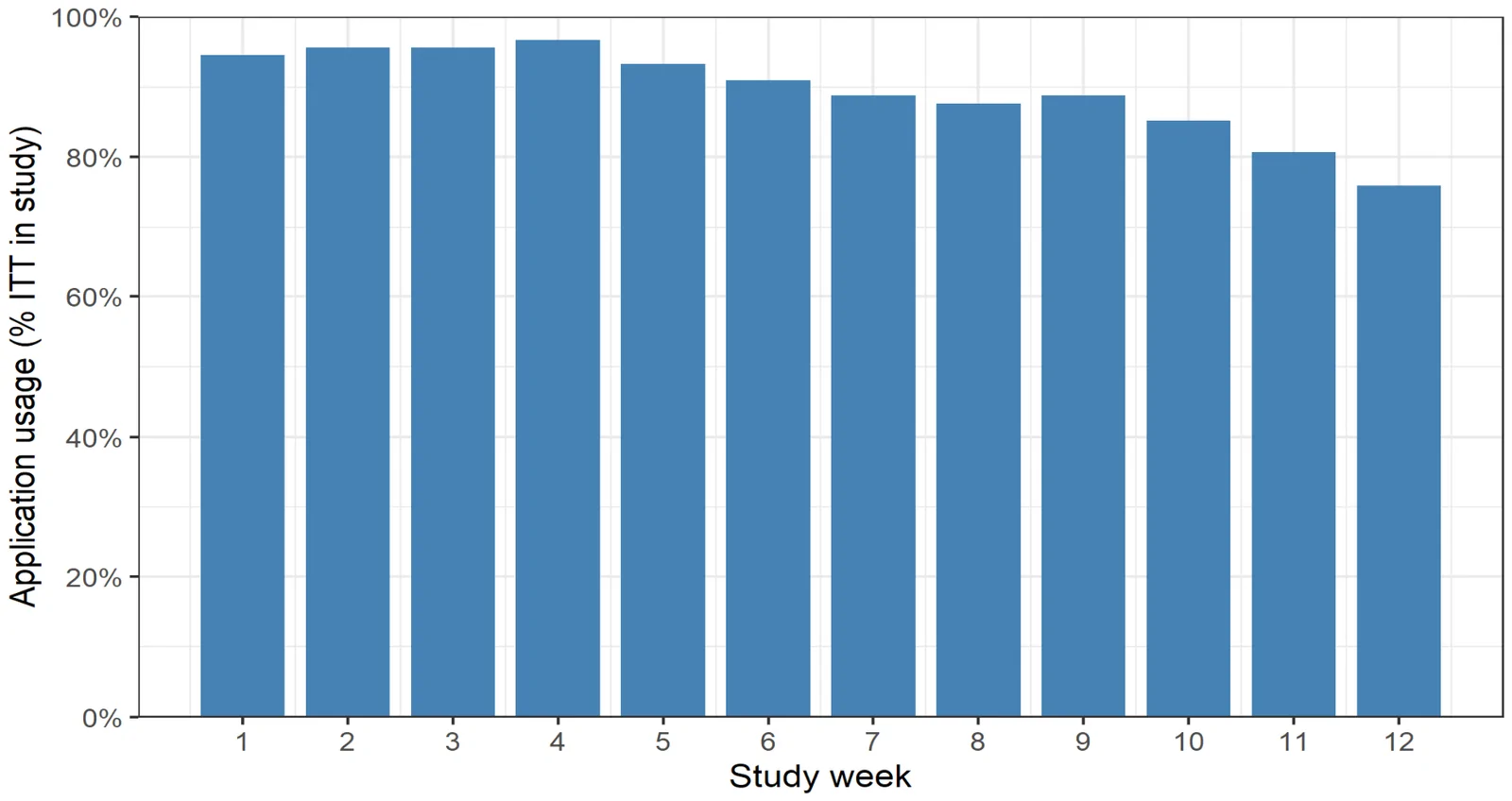
App usage by week in Arm A (ITT). The column “% ITT in study” shows patients with app usage relative to patients in study.

Looking at the use of the app per study week, over 90% of the observable participants used it at least once per week for up to week 6 (this analysis did not break down whether it was used on one or more days per week). In the last week of the study, 75.9 of the observable participants (i.e., who were still participating) still used the app (Figure 4). The average daily usage rate of all participants was 64.9%. Participants between the ages of 40 and 69 showed particularly high adherence, while younger and older people used the app to a lesser extent. However, the data for the latter groups is probably not very meaningful due to the small number of participants per group.

## 4. Discussion

The primary endpoint analysis with the ITT population showed almost no differences in HAE incidence between the app-using intervention group and the control group that did not use the medidux™ app. Also, in sensitivity analysis and analyses of the secondary endpoints “emergency consultations” and “hospitalization” which are a priori related to the occurrence of HAEs [5,6], no significant difference between the two study arms was observed. A limitation of the PRO2 study was the premature termination after 194 patients had been enrolled. This early termination may also have introduced selection bias in how patients were offered study participation across centers. A higher participant number may have led to significant differences between the app use and the control arm. An approximate post-hoc power calculation yielded 10.1% power to detect an effect of this magnitude, consistent with the wide 95% CI observed (0.363–1.752). While the point estimate of the adjusted OR of 0.797 leans in a favorable direction, the study should be interpreted as inconclusive given the limited sample size, low incidence and wide confidence interval. Furthermore, it was assumed that 75% of all patients in the control group would have at least 1 HAE within the study duration of 12 weeks in the sample-size calculation, but this figure was actually only around 20% in both study arms (Table 2). This significantly lower observed HAE incidence also makes it a priori more difficult for an app to have a demonstrably significant effect on adverse events. Nevertheless, the study revealed some interesting findings: participants showed a high level of compliance with the app usage and allowed for the collection of a sufficient amount of data that could be used to analyze various secondary endpoints. The gradual decline in app usage over the observation period may have contributed to underreporting of events; reminder notifications could help sustain adherence in future implementations. It is interesting to note that in both arms almost half of the reported first HAEs occurred within the first 21 days after the start of the therapy and again a significant amount occurred between days 21 and 63. Thereafter only a slow increase in the cumulative incidence was observed until the end of the study. This shows that the chosen observation period of 12 weeks was in principle adequate for analyzing the primary endpoint. Yet it is important to acknowledge that adverse events may also occur beyond this time frame—particularly relevant given that a large proportion of patients received treatment with curative intent. The use of the medidux™ app did not lead to a reduction in CTX/ADC therapy adherence, it was even slightly better in the intervention arm. Furthermore, no app-related increase in CTX dose reduction was observed. The symptoms documented by the participants in arm A via the medidux™ app correspond well with the expected side effects of combined targeted HER2 + chemotherapy or ADC therapy, with fatigue and diarrhea occurring most frequently. The heterogeneity of regimens received by participants should be noted, as chemotherapy combined with HER2-targeted antibodies and antibody-drug conjugates (ADCs) are associated with distinct toxicity profiles. As the app’s severity scale and its mapping to severity grades are application-specific and not formally validated, self-reported symptom grades are not directly comparable to clinician-rated assessments; the primary endpoint was unaffected, as it was based on investigator-documented and independently adjudicated events.

The number of other studies with cancer patients that have investigated the influence of a “health app” on the incidence of medically “hard endpoints” (such as hospitalizations, adverse events, emergency consultations, survival endpoints) is limited. Basch et al. were among the first to investigate the influence of an app on the health of cancer patients [7,8]. The primary objective in the study was the web-based QoL self-assessment by the patients with metastatic solid tumors (breast, urogenital, gynecological, or lung cancer) treated with chemotherapy after SoC. As secondary endpoints, positive effects of the web-based QoL self-assessment by the patient on the number of emergency admissions, hospitalizations and survival time were shown. However, this involved intensive individual support by specially trained staff (nurses). In a study with patients with various tumor entities (including breast cancer) who received approved oral cancer drugs, a reduction in hospitalization and treatment-related toxicities ≥3 CTCAE was observed when using a web portal-smartphone app portal to report symptoms [9]. In this study, the intensive individual guidance of the patients by specially trained staff (nurses) was also part of the study design. In a study of patients with non-metastatic breast cancer, colorectal cancer, Hodgkin’s disease or non-Hodgkin’s lymphoma who were receiving adjuvant chemotherapy, a significant reduction in symptom burden was observed in the intervention group, which was monitored remotely using a digital application [14]. An improvement in physical well-being, also based on patient self-assessment, was observed in a study of patients with colorectal, breast or gynecological cancer who received chemotherapy [15]. In the PRECYCLE study with clearly defined patient cohorts, patients with hormone receptor-positive, HER2-negative locally advanced or metastatic breast cancer who received palbociclib and an aromatase inhibitor or palbociclib and fulvestrant, a significantly more positive effect on self-assessed quality of life was found in the intervention group using a “full version” of an app designed for breast cancer patients than in the control group using an app version with limited functionality [10]. A secondary endpoint in the PRECYCLE study analyzed the incidence of AEs and SAEs in both study arms. While the overall distribution of AEs was similar in each arm, patients in the intervention arm had a favorable hazard ratio of 0.67 (95% CI 0.46–0.97; *p* = 0.04) for time to first SAE and were significantly less likely to experience an SAE overall than patients in the control arm [11]. In a three-arm study of patients with a diagnosis of ductal carcinoma in situ or HR-positive stage I to III breast cancer and adjuvant endocrine therapy, including aromatase inhibitors or tamoxifen, while there was no difference in symptom burden between the groups receiving apps with different functionality and support from the study teams, hospitalizations and emergency visits were reduced in the app-based remote monitoring groups [16].

An objective comparison of the results of these studies with the results of the PRO2 study is made more difficult by the fact that different app and web applications were of course used, a much broader patient population with different cancer entities was often included in the studies and the app application was sometimes supported by intensive monitoring and advice from specially trained study staff [7,8,9,15]. Unlike Basch et al. [7,8], the CAPRI trial [9], and PRECYCLE [10,11], the medidux™ app used in PRO2 did not include systematic review of the self-reported symptom data by the treating team, nor a predefined clinical response to alerts—patients were merely prompted to contact the care team themselves if symptoms fell outside the acceptable range. This absence of an integrated clinical feedback loop may partly explain why the intervention did not translate into a measurable reduction in HAEs, and the positive effect of the app may consequently have been underestimated. Whether patients acted on these prompts and which clinical actions followed were not systematically recorded, which limits the extent to which intervention uptake and the mechanism underlying a potential app effect can be assessed in this trial. Interventions and interactions with healthcare teams triggered by PRO-driven alerts appear to have the potential to reduce adverse events [17], whereas less structured symptom monitoring has shown no such benefit [18], indicating that further studies are needed to determine the optimal design of app-based interventions using PROs. All these studies examined the patient’s self-assessed well-being as a primary endpoint, while “hard endpoints” documented by healthcare professionals were then secondary endpoints at best. A key difference between PRO2 and the other studies considered in this discussion is the use of a blinded adjudicator whose independent assessment of the severity of HAEs, the primary endpoint of the study, was decisive, which also helped reduce the risk of bias related to the corresponding author’s role in the company developing medidux™. However, some secondary clinical data such as hospitalizations and emergency consultations were assessed by the “unblinded” participating investigators.

The post hoc analysis of various studies with patients with HER2-positive breast cancer showed that the patients’ self-assessment corresponds very well with overall survival and progression-free survival and the occurrence of grade 3 adverse events [19]. Why there was no significant effect of the medidux™ app used in the PRO2 on the “hard” clinical endpoints HAE incidence, hospitalization and emergency consultations cannot be conclusively explained, regardless of the limitation due to insufficient recruitment. One aspect that may play a role is the incidence of AEs, which is related to the stage of cancer. Some of the studies in which the apps used influenced clinical parameters included partially or exclusively metastatic cancer patients [5,7,8,11], who generally have a higher incidence of AEs. In contrast, the AE incidence observed in patients with HER2-positive primary breast cancer investigated in PRO2 was lower than originally assumed. A lower app effect may be related to a low AE incidence. The collected data will be evaluated in a subsequent publication using machine learning regarding the occurrence of unplanned consultations and hospitalizations.

## 5. Conclusions

Although the effect on HAE rates was not significant, intensive app use was observed, and adverse events recorded by participants corresponded well with the known drug side-effect profiles. These exploratory findings suggest a potential supporting role of the app during out-patient cancer treatment, which needs confirmation in adequately powered studies. The results indicate that the medidux™ app may be suitable as a therapy diary for standardized, structured, and dynamic recording of symptoms, potentially supporting communication between patients and doctors/study teams. Application in decentralized studies and registries appears promising and should be further studied.

## Figures and Tables

**Table 2:** Incidence of HAEs (CTCAE severity ≥3).

Parameter	Level	Statistic	Arm A	Arm B	Total
HAE Incidence	(all)	N (missing)	87 (6)	93 (3)	180 (9)
	0	N (%)	72 (82.76%)	73 (78.49%)	145 (80.56%)
	1	N (%)	15 (17.24%)	20 (21.51%)	35 (19.44%)

**Table 3:** Logistic regression of HAE incidence, effect estimates (reference-based multiple imputation).

	Estimate	Lower 95% CI	Upper 95% CI	*p*-Value
Odds Ratio Arm (A/B)	0.797	0.363	1.752	0.569
Proportion Arm B	0.213	0.141	0.309	
Proportion Arm A	0.174	0.107	0.270	

**Table 4:** Count of emergency consultations and hospitalizations (ITT).

Parameter	Level	Statistic	Arm B	Arm A	Total
Emergency Consultations	(all)	N (missing)	96 (0)	93 (0)	189 (0)
	0	N (%)	69 (71.88%)	71 (76.34%)	140 (74.07%)
	1	N (%)	15 (15.62%)	16 (17.2%)	31 (16.4%)
	2	N (%)	10 (10.42%)	3 (3.23%)	13 (6.88%)
	3	N (%)	1 (1.04%)	1 (1.08%)	2 (1.06%)
	4	N (%)	0 (0%)	1 (1.08%)	1 (0.53%)
	5	N (%)	1 (1.04%)	1 (1.08%)	2 (1.06%)
Hospitalizations	(all)	N (missing)	96 (0)	93 (0)	189 (0)
	0	N (%)	81 (84.38%)	84 (90.32%)	165 (87.3%)
	1	N (%)	11 (11.46%)	8 (8.6%)	19 (10.05%)
	2	N (%)	3 (3.12%)	1 (1.08%)	4 (2.12%)
	3	N (%)	1 (1.04%)	0 (0%)	1 (0.53%)

## Data Availability

The original contributions presented in this study are included in the article/Supplementary Material. Further inquiries can be directed to the corresponding author.

## Supplementary Materials

The following supporting information can be downloaded at: https://www.mdpi.com/article/10.3390/curroncol33100618/s1, Figure S1: Therapy adherence; Table S1: Observation duration; Table S2: HAE incidence by system organ class and preferred term; Table S3: Incidence of HAEs (PP population); Table S4: Incidence of Serious Adverse Events; Table S5: Logistic regression of SAE incidence, effect estimates; Table S6: Incidence proportion with chemotherapy or antibody-drug-conjugate dose reductions; Table S7: Logistic regression of dose reduction incidence, effect estimates; Table S8: Negative binomial regression, emergency consultation counts; Table S9: Negative binomial regression, hospitalization.

## Abbreviations

The following abbreviations are used in this manuscript:

ADC	Antibody-Drug Conjugate
AE	Adverse Events
CI	Confidence Interval
CTCAE	Common Terminology Criteria for Adverse Events
CTX	Chemotherapy
ECOG	Eastern Cooperative Oncology Group
eCRF	electronic Case Report Form
ePRO	electronic Patient-Reported Outcomes
HAE	high-grade AEs (defined as AEs with CTCAE severity ≥3 and SAEs)
HER2	Human Epidermal growth factor Receptor 2
IHC	Immunohistochemistry
ISH	In situ Hybridization
ITT	Intention to Treat
MedDRA	Medical Dictionary for Regulatory Activities
NCI	National Cancer Institute
PD	Protocol Deviation
PP	Per Protocol
SAE	Serious Adverse Events
SoC	Standard of Care
TKI	Tyrosine Kinase Inhibitor

## References

[1] Gnanasakthy A., DeMuro C., Clark M., Haydysch E., Ma E., Bonthapally V. (2016). Patient-Reported Outcomes Labeling for Products Approved by the Office of Hematology and Oncology Products of the US Food and Drug Administration (2010–2014). J. Clin. Oncol..

[2] Henry D.H., Viswanathan H.N., Elkin E.P., Traina S., Wade S., Cella D. (2008). Symptoms and treatment burden associated with cancer treatment: Results from a cross-sectional national survey in the U.S. Support. Care Cancer.

[3] Prince R.M., Atenafu E.G., Krzyzanowska M.K. (2015). Hospitalizations During Systemic Therapy for Metastatic Lung Cancer: A Systematic Review of Real World vs Clinical Trial Outcomes. JAMA Oncol..

[4] Whitney R.L., Bell J.F., Tancredi D.J., Romano P.S., Bold R.J., Wun T., Joseph J.G. (2019). Unplanned Hospitalization Among Individuals With Cancer in the Year After Diagnosis. J. Oncol. Pract..

[5] Brooks G.A., Abrams T.A., Meyerhardt J.A., Enzinger P.C., Sommer K., Dalby C.K., Uno H., Jacobson J.O., Fuchs C.S., Schrag D. (2014). Identification of potentially avoidable hospitalizations in patients with GI cancer. J. Clin. Oncol..

[6] Foltran L., Aprile G., Pisa F.E., Ermacora P., Pella N., Iaiza E., Poletto E., Lutrino S.E., Mazzer M., Giovannoni M. (2014). Risk of unplanned visits for colorectal cancer outpatients receiving chemotherapy: A case-crossover study. Support. Care Cancer.

[7] Basch E., Deal A.M., Kris M.G., Scher H.I., Hudis C.A., Sabbatini P., Rogak L., Bennett A.V., Dueck A.C., Atkinson T.M. (2016). Symptom Monitoring With Patient-Reported Outcomes During Routine Cancer Treatment: A Randomized Controlled Trial. J. Clin. Oncol..

[8] Basch E., Deal A.M., Dueck A.C., Scher H.I., Kris M.G., Hudis C., Schrag D. (2017). Overall Survival Results of a Trial Assessing Patient-Reported Outcomes for Symptom Monitoring During Routine Cancer Treatment. JAMA.

[9] Mir O., Ferrua M., Fourcade A., Mathivon D., Duflot-Boukobza A., Dumont S., Baudin E., Delaloge S., Malka D., Albiges L. (2022). Digital remote monitoring plus usual care versus usual care in patients treated with oral anticancer agents: The randomized phase 3 CAPRI trial. Nat. Med..

[10] Harbeck N., Fasching P.A., Wuerstlein R., Degenhardt T., Lüftner D., Kates R.E., Schumacher J., Räth P., Hoffmann O., Lorenz R. (2023). Significantly longer time to deterioration of quality of life due to CANKADO PRO-React eHealth support in HR+ HER2- metastatic breast cancer patients receiving palbociclib and endocrine therapy: Primary outcome analysis of the multicenter randomized AGO-B WSG PreCycle trial. Ann. Oncol..

[11] Harbeck N., Kates R., Schinköthe T., Schumacher J., Wuerstlein R., Degenhardt T., Lüftner D., Räth P., Hoffmann O., Lorenz R. (2023). Favorable impact of therapy management by an interactive eHealth system on severe adverse events in patients with hormone receptor-positive, HER2-negative locally advanced or metastatic breast cancer treated by palbociclib and endocrine therapy. Cancer Treat. Rev..

[12] Trojan A., Kühne C., Kiessling M., Schumacher J., Dröse S., Singer C., Jackisch C., Thomssen C., Kullak-Ublick G.A. (2024). Impact of Electronic Patient-Reported Outcomes on Unplanned Consultations and Hospitalizations in Patients With Cancer Undergoing Systemic Therapy: Results of a Patient-Reported Outcome Study Compared With Matched Retrospective Data. JMIR Form. Res..

[13] Trojan A., Leuthold N., Thomssen C., Rody A., Winder T., Jakob A., Egger C., Held U., Jackisch C. (2021). The Effect of Collaborative Reviews of Electronic Patient-Reported Outcomes on the Congruence of Patient- and Clinician-Reported Toxicity in Cancer Patients Receiving Systemic Therapy: Prospective, Multicenter, Observational Clinical Trial. J. Med. Internet Res..

[14] Maguire R., McCann L., Kotronoulas G., Kearney N., Ream E., Armes J., Patiraki E., Furlong E., Fox P., Gaiger A. (2021). Real time remote symptom monitoring during chemotherapy for cancer: European multicentre randomised controlled trial (eSMART). BMJ.

[15] Absolom K., Warrington L., Hudson E., Hewison J., Morris C., Holch P., Carter R., Gibson A., Holmes M., Clayton B. (2021). Phase III Randomized Controlled Trial of eRAPID: eHealth Intervention During Chemotherapy. J. Clin. Oncol..

[16] Graetz I., Hu X., Kocak M., Krukowski R.A., Anderson J.N., Waters T.M., Curry A.N., Robles A., Paladino A., Stepanski E. (2024). Remote Monitoring App for Endocrine Therapy Adherence Among Patients With Early-Stage Breast Cancer: A Randomized Clinical Trial. JAMA Netw. Open.

[17] Karsten M.M., Gebert P., Pross T., Hage A.M., Dordevic A.D., Stephan S., Kühn F., Doppelbauer L., Blohmer J.-U., Fischer F. (2026). Electronic Patient-Reported Outcome Monitoring with Alert-Based Interventions in Metastatic Breast Cancer: A Randomized Clinical Trial. JAMA Oncol..

[18] Okuyama H., Takada F., Taira N., Nakamura S. (2024). A randomized trial of the impact of symptom monitoring using an electronic patient-reported outcome app on health-related quality of life in postmenopausal breast cancer patients receiving adjuvant endocrine therapy. Breast Cancer.

[19] Modi N.D., Danell N.O., Perry R.N.A., Abuhelwa A.Y., Rathod A., Badaoui S., McKinnon R.A., Haseloff M., Shahnam A., Swain S.M. (2022). Patient-reported outcomes predict survival and adverse events following anticancer treatment initiation in advanced HER2-positive breast cancer. ESMO Open.

