# Supplementary material for: Incidence Rates of Adverse Events (CTCAE Severity ≥3) During HER2-Directed Breast Cancer Treatment Assessing the Impact of App-Based Electronic Patient-Reported Outcomes in the Randomized Controlled PRO2 Trial
Source: Curr Oncol. 2026 Oct 14;33(10):618. doi: 10.3390/curroncol33100618 (PMC13703651; doi:10.3390/curroncol33100618)
Supplement: Supplementary file 1 [file curroncol-33-00618-s001.zip › curroncol-4493235-supplementary.pdf]

Figure S1. Therapy adherence.

Figure 5.2.2 PP Population

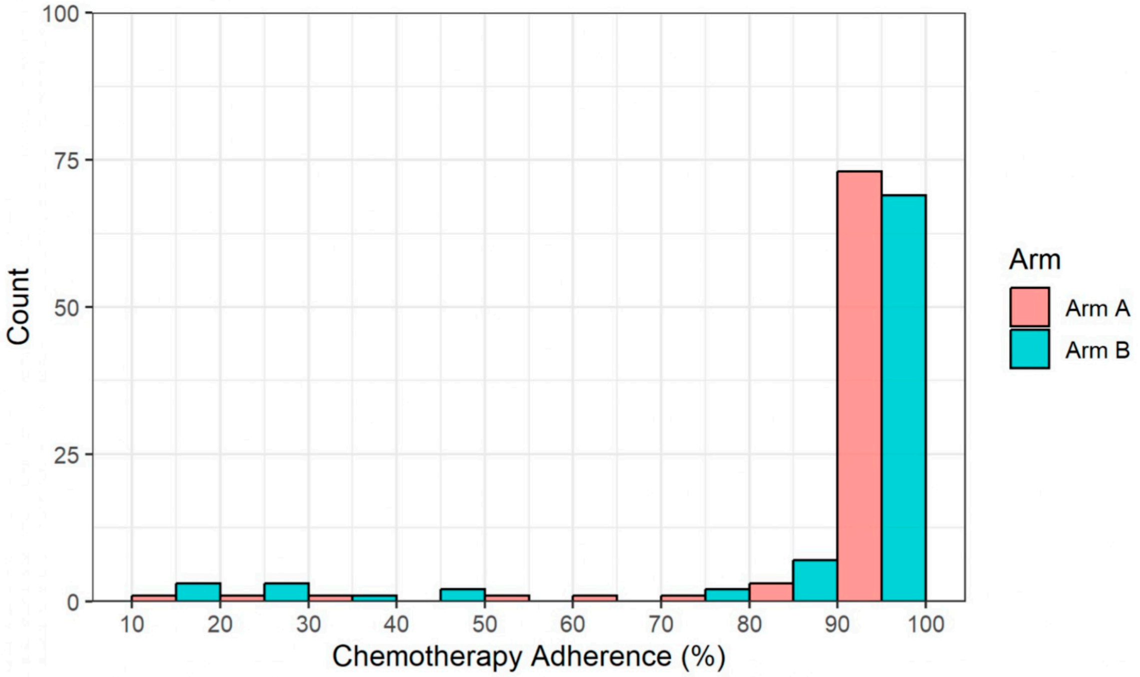

Histogram of estimated Chemotherapy Adherence in percent.

Table S1. Observation duration.

Duration of observation in weeks.

Parameter	Level	Statistic	Arm A	Arm B	Total
Observation duration	(all)	N (missing)	93 (0)	96 (0)	189 (0)
		Mean	12.01	12.47	12.24
		Std	2.475	1.381	2.004
		Min	0	3.4	0
		Q1	12.1	12.1	12.1
		Median	12.1	12.1	12.1
		Q3	12.6	13.03	13
		Max	15.3	16	16
		IQR	0.5	0.925	0.9

Table S2. HAE incidence by system organ class and preferred term.

HAE incidence by system organ class and preferred term (ITT, no imputation)

System Organ Class	Preferred Term	Arm A (n=93)	Arm A (%)	Arm B (n=96)	Arm B (%)	Total (n=189)	Total (%)
Blood and lymphatic system disorders	(all)	4	4.30	3	3.12	7	3.70
	Anaemia	1	1.08	2	2.08	3	1.59
	Febrile neutropenia	2	2.15	1	1.04	3	1.59
	Leukopenia	1	1.08	0	0.00	1	0.53
	Thrombocytopenia	0	0.00	1	1.04	1	0.53
Ear and labyrinth disorders	(all)	0	0.00	1	1.04	1	0.53
	Vertigo	0	0.00	1	1.04	1	0.53
Gastrointestinal disorders	(all)	2	2.15	11	11.46	13	6.88
	Diarrhoea	1	1.08	7	7.29	8	4.23
	Duodenal ulcer	1	1.08	0	0.00	1	0.53
	Gastritis	0	0.00	1	1.04	1	0.53
	Gastritis erosive	0	0.00	1	1.04	1	0.53
	Nausea	0	0.00	1	1.04	1	0.53
	Stomatitis	0	0.00	1	1.04	1	0.53
	Vomiting	0	0.00	1	1.04	1	0.53
General disorders and administration site conditions	(all)	3	3.23	4	4.17	7	3.70
	Chest pain	1	1.08	0	0.00	1	0.53
	Fatigue	0	0.00	3	3.12	3	1.59
	General physical health deterioration	1	1.08	0	0.00	1	0.53
	Malaise	0	0.00	1	1.04	1	0.53
	Non-cardiac chest pain	1	1.08	0	0.00	1	0.53
Immune system disorders	(all)	1	1.08	0	0.00	1	0.53
	Hypersensitivity	1	1.08	0	0.00	1	0.53
Infections and infestations	(all)	6	6.45	3	3.12	9	4.76

System Organ Class	Preferred Term	Arm A (n=93)	Arm A (%)	Arm B (n=96)	Arm B (%)	Total (n=189)	Total (%)
	COVID-19	1	1.08	0	0.00	1	0.53
	Device related infection	1	1.08	0	0.00	1	0.53
	Enterocolitis infectious	1	1.08	0	0.00	1	0.53
	Infection	1	1.08	1	1.04	2	1.06
	Influenza	0	0.00	1	1.04	1	0.53
	Pneumonia	1	1.08	0	0.00	1	0.53
	Skin infection	1	1.08	0	0.00	1	0.53
	Urinary tract infection	0	0.00	1	1.04	1	0.53
Investigations	(all)	2	2.15	1	1.04	3	1.59
	Alanine aminotransferase increased	0	0.00	1	1.04	1	0.53
	Neutrophil count decreased	2	2.15	0	0.00	2	1.06
Metabolism and nutrition disorders	(all)	0	0.00	1	1.04	1	0.53
	Dehydration	0	0.00	1	1.04	1	0.53
Neoplasms benign, malignant and unspecified (incl cysts and polyps)	(all)	0	0.00	1	1.04	1	0.53
	Skin cancer	0	0.00	1	1.04	1	0.53
Nervous system disorders	(all)	0	0.00	1	1.04	1	0.53
	Dizziness	0	0.00	1	1.04	1	0.53
Psychiatric disorders	(all)	0	0.00	1	1.04	1	0.53
	Depression	0	0.00	1	1.04	1	0.53
Renal and urinary disorders	(all)	1	1.08	2	2.08	3	1.59
	Acute kidney injury	0	0.00	1	1.04	1	0.53
	Urinary retention	1	1.08	1	1.04	2	1.06
Skin and subcutaneous tissue disorders	(all)	0	0.00	1	1.04	1	0.53
	Eczema	0	0.00	1	1.04	1	0.53

Table S3. Incidence of HAEs (PP population).

Incidence of high-grade Adverse Events (PP, no imputation)

Parameter	Level	Statistic	Arm B	Arm A	Total
HAE Incidence	(all)	N (missing)	87 (0)	82 (0)	169 (0)
	0	N (%)	69 (79.31%)	67 (81.71%)	136 (80.47%)
	1	N (%)	18 (20.69%)	15 (18.29%)	33 (19.53%)

Table S4. Incidence of Serious Adverse Events.

Incidence of Serious Adverse Events (ITT, no imputation)

Parameter	Level	Statistic	Arm B	Arm A	Total
SAE Incidence	(all)	N (missing)	93 (3)	87 (6)	180 (9)
	0	N (%)	78 (83.87%)	78 (89.66%)	156 (86.67%)
	1	N (%)	15 (16.13%)	9 (10.34%)	24 (13.33%)

Table S5. Logistic regression of SAE incidence, effect estimates.

Logistic Regression of SAE Incidence, Wald Statistics (reference-based multiple imputation)

	Estimate	Std	P-Value	Lower 95% CI	Upper 95% CI
(Intercept)	-5.705	1.351	<0.001	-8.372	-3.038
Age	0.071	0.023	0.002	0.027	0.116
Treatment intention – Palliative (vs Curative)	0.924	0.649	0.156	-0.357	2.204
Therapy type – ADC (vs CTX)	-1.144	1.217	0.349	-3.547	1.260
Study arm – Arm A (vs B)	-0.452	0.470	0.337	-1.380	0.475

Table S6. Incidence proportion with chemotherapy or antibody-drug-conjugate dose reductions.

Incidence of CTX dose reductions (PP, no imputation)

Parameter	Level	Statistic	Arm B	Arm A	Total
Dose Reductions	(all)	N (missing)	87 (0)	82 (0)	169 (0)
	0	N (%)	80 (91.95%)	72 (87.8%)	152 (89.94%)
	1	N (%)	7 (8.05%)	10 (12.2%)	17 (10.06%)

Table S7. Logistic regression of dose reduction incidence, effect estimates.

Logistic Regression of Dose Reduction Incidence (ITT), Wald Statistics (Arm-MAR multiple imputation)

	Estimate	Std	P-Value	Lower 95% CI	Upper 95% CI
(Intercept)	-6.587	1.622	<0.001	-9.789	-3.385
Age	0.074	0.026	0.006	0.022	0.127
Therapy type – ADC (vs CTX)	-0.339	1.177	0.774	-2.662	1.985
Study arm – Arm A (vs B)	0.508	0.541	0.349	-0.560	1.575

Table S8. Negative binomial regression, emergency consultation counts; Table S9: Negative binomial regression, hospitalization.

Negative binomial regression of emergency visit counts (ITT)

	Estimate	Std	Z-Value	P-Value	Lower 95% CI	Upper 95% CI
(Intercept)	-5.423	0.786	-6.903	<0.001	-6.973	-3.966
Study arm – Arm A (vs B)	-0.172	0.299	-0.574	0.566	-0.767	0.420
Age	0.039	0.014	2.845	0.004	0.014	0.066
Treatment intention – Palliative (vs Curative)	-0.282	0.521	-0.541	0.589	-1.374	0.737
Therapy type – ADC (vs CTX)	-0.171	0.717	-0.238	0.812	-1.641	1.216

θ = 0.649 (SE = 0.251); null deviance = 142.53 on 187 df; residual deviance = 132.89 on 183 df; AIC = 321.80; 0 obs deleted.

Table S9. Negative binomial regression, hospitalization.

Negative binomial regression of hospitalization counts (ITT)

	Estimate	Std	Z-Value	P-Value	Lower 95% CI	Upper 95% CI
(Intercept)	-8.380	1.211	-6.922	<0.001	-11.029	-6.061
Study arm – Arm A (vs B)	-0.553	0.431	-1.284	0.199	-1.439	0.284
Age	0.075	0.020	3.816	<0.001	0.036	0.118
Treatment intention – Palliative (vs Curative)	0.348	0.621	0.560	0.575	-0.984	1.525
Therapy type – ADC (vs CTX)	-1.298	1.249	-1.039	0.299	-4.394	0.736

θ = 0.955 (SE = 0.825); null deviance = 105.72 on 187 df; residual deviance = 86.72 on 183 df; AIC = 171.91; 0 obs deleted.
